# The Minimal Deneddylase Core of the COP9 Signalosome Excludes the Csn6 MPN^−^ Domain

**DOI:** 10.1371/journal.pone.0043980

**Published:** 2012-08-30

**Authors:** Elah Pick, Amnon Golan, Jacob Z. Zimbler, Liquan Guo, Yehonatan Sharaby, Tomohiko Tsuge, Kay Hofmann, Ning Wei

**Affiliations:** 1 Department of Biology, University of Haifa at Oranim, Tivon, Israel; 2 Department of Evolutionary and Environmental Biology, University of Haifa, Haifa, Israel; 3 Department of Molecular, Cellular and Developmental Biology, Yale University, New Haven, Connecticut, United States of America; 4 Institute for Genetics, University of Cologne, Cologne, Germany; University of Minnesota, United States of America

## Abstract

The COP9 signalosome (CSN) is a eukaryotic protein complex, which regulates a wide range of biological processes mainly through modulating the cullin ubiquitin E3 ligases in the ubiquitin-proteasome pathway. The CSN possesses a highly conserved deneddylase activity that centers at the JAMM motif of the Csn5 subunit but requires other subunits in a complex assembly. The classic CSN is composed of 8 subunits (Csn1–8), yet in several *Ascomycota*, the complex is smaller and lacks orthologs for a few CSN subunits, but nevertheless contains a conserved Csn5. This feature makes yeast a powerful model to determine the minimal assemblage required for deneddylation activity. Here we report, that Csi1, a diverged *S. cerevisiae* CSN subunit, displays significant homology with the carboxyl terminal domain of the canonical Csn6, but lacks the amino terminal MPN^-^ domain. Through the comparative and experimental analyses of the budding yeast and the mammalian CSNs, we demonstrate that the MPN^−^ domain of the canonical mouse Csn6 is not part of the CSN deneddylase core. We also show that the carboxyl domain of Csn6 has an indispensable role in maintaining the integrity of the CSN complex. The CSN complex assembled with the carboxyl fragment of Csn6, despite its lack of an MPN^−^ domain, is fully active in deneddylation of cullins. We propose that the budding yeast Csi1 is a functional equivalent of the canonical Csn6, and thus the composition of the CSN across phyla is more conserved than hitherto appreciated.

## Introduction

The COP9 signalosome (CSN) is an evolutionarily conserved protein complex with a canonical composition of eight subunits (Csn1–8) [1]. The most studied biochemical activity of the CSN is hydrolysis of the ubiquitin-like protein Nedd8/Rub1 from the cullin proteins (deneddylation or derubbylation). Cullins are the scaffold components of cullin-RING ligase (CRL) protein complexes, which belong to the largest family of ubiquitin E3 ligases in the cell [2], [3], [4]. Deneddylation of cullins is necessary for maintaining the stability and the sustained activity of CRLs in vivo, allowing the ligases to polyubiquitinate a large number of substrates that are targeted by the ubiquitin-proteasome system [3], [5], [6], [7].

The CSN belongs to a family of protein complexes known as the PCI complexes, which include the lid subcomplex of the 26S proteasome, the CSN, and the eukaryotic translation initiation factor-3 (eIF3) (Table 1, [8]). Members of this family play key roles in the regulation of protein life span from translation to degradation [8], [9], [10]. Subunits of these complexes share large structural elements such as PCI or MPN (Mpr1/Pad1 N-terminal) domains [11], and are arranged in a comparable architecture [12]. The PCI domain, which is found in six subunits of each complex, serves as a structural scaffold that supports complex integrity via interactions between subunits [11], [13], [14]. All three complexes also contain a pair of MPN domain-containing subunits. In some cases, the MPN domain harbors a JAMM (JAB1-MPN-MOV34) metal-binding motif, which is the catalytic center of the CSN’s deneddylase activity [15], [16], [17]. Here we refer to the JAMM-containing MPN domain as the “MPN^+^” domain, while the MPN domain that lacks the JAMM catalytic motif is referred to as the “MPN^−^” domain (Table 1).

**Table 1:** Subunits comparison of related MPN-containing complexes in human and yeast.

Species	*H. sapiens*					*S. cerevisiae*		
ComplexDomain	BRISC	BRCA1-A	eIF3	lid	CSN	eIF3	lid	CSN
**MPN^−^**	ABRO1	ABRAXAS	eIF3f eIF3h	MOV34/RPN8	CSN6/hVIP	(−)	Rpn8	(−)
**MPN^+^/JAMM**	BRCC36	BRCC36	(−)	POH1/RPN11	CSN5/JAB1	(−)	Rpn11	Csn5/Rri1
**PCI**	(−)	(−)	eIF3e eIF3c eIF3m eIF3a eIF3l eIF3k	PSMD12PSMD13PSMD11PSMD6PSMD3 PSMD8	CSN1CSN2CSN3CSN4CSN7CSN8	eIF3ceIF3a	Rpn3Rpn4Rpn5Rpn6Rpn7Rpn9	Csn9Csn10Csn11Rpn5
**other**	BRCC45/BREMERIT40/NBA	RAP80 BRCC45/BREMERIT40/NBA	eIF3g eIF3deIF3b eIF3ieIF3j	RPN15/DSS1	(−)	eIF3geIF3beIF3ieIF3j	Rpn15/Sem1	Csi1

Human BRISC BRCA1-A, complexes are deubiquitinating enzymes. CSN is a deneddylase complex. The proteasome lid is a deubiquitinating enzyme when integrated into the proteasome. The eukaryotic translation initiation factor-3 (eIF3) complex has not been found to have a isopeptidase activity.

MPN proteins are also found in complexes without PCI proteins, such as the BRISC and BRCA1-A deubiquitinating (DUB) complexes, both of which are absent in the *S. cerevisiae* genome (Table 1) [18]. The MPN^+^ proteins contain the JAMM-mediated isopeptidase activity center, essential to the deubiquitinating activity of the proteasome, BRISC and BRCA1-A, or the deneddylation activity of the CSN [2], [15], [17], [19]. At least with the lid and the CSN, the enzymatic activity requires an integrated multi-subunit complex. Interestingly, the MPN^+^ proteins tend to exist in pairs with an MPN^−^ subunit in these complexes (Table 1). Little is known about whether the MPN^−^ domain contributes to the enzymatic activity of the complexes, although there have been speculations that the MPN^−^ domain might have a role in the JAMM-dependent activity [20].

Csn6 is the MPN^−^ subunit in the CSN, and it has been speculated to play a role in structural integrity of the complex [21], [22]. Recent studies have linked Csn6 to tumorigenesis via the MDM2-p53 signaling pathway in conjunction with COP1 and 14-3-3σ [23], [24]. Interestingly, Csn6 is being trimmed by caspases during apoptosis [25], [26]. Still, experimental evidence on whether Csn6 or its MPN^−^ domain contributes to the integrity or the deneddylase activity of the CSN is lacking.

**Figure 1:**
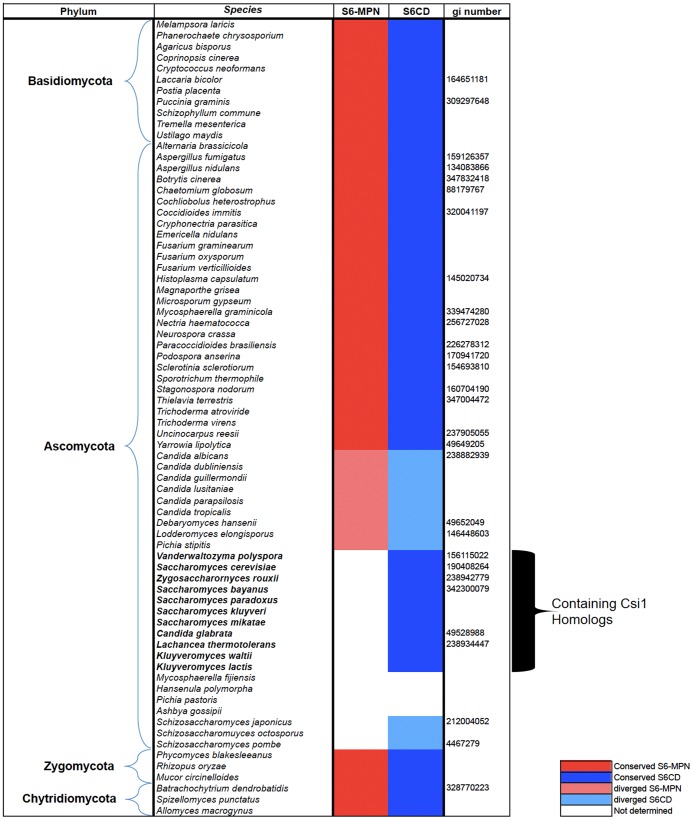
Bioinformatic identification of two distinct domains within Csn6 (S6MPN and S6CD) in various fungal species. The Csn6-like proteins from fungal species were identified from multiple genome databases. Species were grouped according to their phyla, and their conserved (dark red/blue) or diverged (light red/blue) domains, MPN and S6CD. White boxes indicate the missing domains. The data is based on the “fungal genome program” of the “DOE Joint Genome Institute” (http://genome.jgi-psf.org/programs/fungi/index.jsf) and the “fungal genome initiative” of the Broad Institute (http://www.broadinstitute.org/scientific-community/science/projects/fungal-genome-initiative/fungal-genome-initiative).

**Figure 2:**
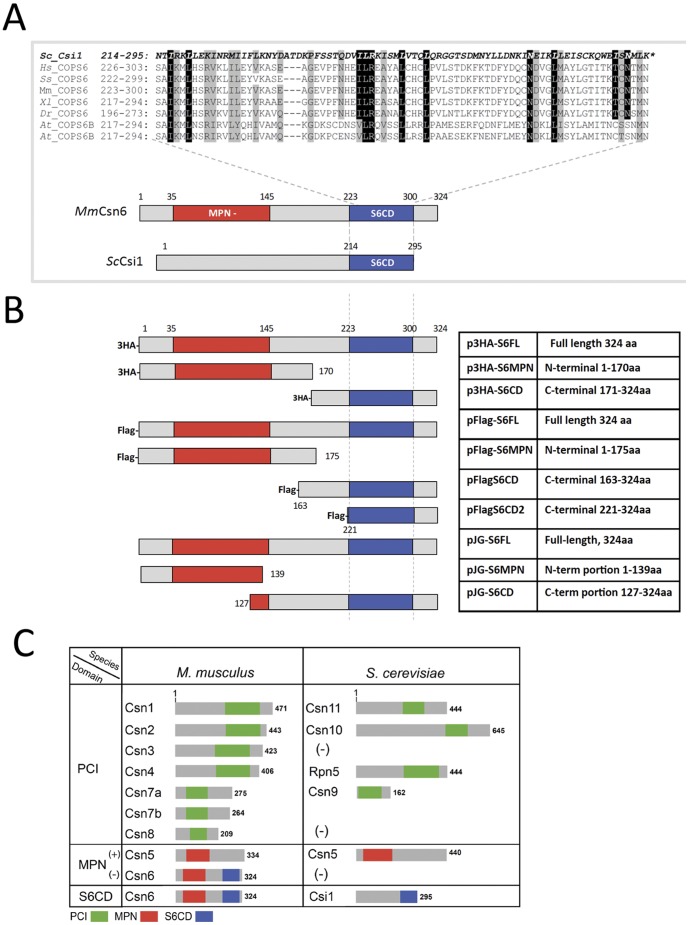
Correlation and homology between the Csi1 subunit of *Sc*CSN with the Csn6 subunit of canonical CSN complex. **A:** A schematic representation highlighting amino acid sequence alignment at the S6CD domain between that from Csi1 of *S. cerevisiae (Sc)* and that from Csn6 (COPS6) of human (*Hs*), mouse (*Mm*), porcine (*Ss*), Xenopus (Xl), Drosophila (Dr), Arabidopsis (At). **B:** A schematic diagram of Csn6 constructs that have been used in this study: FL, full length; S6MPN, MPN^−^ domain; S6CD, C terminal domain; S6CD2, a short version of S6CD. Exact length of truncations is summarized in the table on the right. **C:** A diagram showing the corresponding relationships between subunits of mouse (*M. musculus*) CSN and those of budding yeast (*S. cerevisiae*) CSN.

Although CSN’s deneddylase activity is highly conserved, its subunit composition varies in several unicellular organisms [12]. Specifically, orthologs of Csn6 and Csn8 are frequently missing in lower organisms such as many fungi species where non-canonical CSN complexes exist (Figure 1) [27], [28], [29]. In the budding yeast *S. cerevisiae*, the CSN contains four PCI subunits (Csn9, Csn10/Rri2, Csn11/Pci8, Rpn5), one MPN^+^ subunit (Csn5/Rri1), and Csi1, a unique subunit that contains neither MPN nor PCI recognition domains (Table 1, Figure 2C) [19], [29]. Using the budding yeast CSN as the starting point, we aimed to define the core composition of the CSN that is required for its deneddylase activity, and to comprehend Csi1 with regard to its link to canonical CSN subunits, functionally or bioinformatically.

Here we show that Csi1 displays significant amino acid sequence homology with the carboxyl terminal domain of Csn6 (S6CD), but not with the characteristic MPN^−^ domain. We also demonstrate that a mammalian Csn6 truncation mutant containing S6CD, but not the MPN^−^ domain, is sufficient for assembly of the CSN complex that is fully active in deneddylating multiple cullins. This led us to conclude that the MPN^−^ domain, which is missing in CSN complexes of multiple yeast species, is not a prerequisite for the deneddylase activity of the CSN.

## Results

### Identification of the S6CD Domain and the Homologs of Csn6

The budding yeast CSN (*Sc*CSN) consists of six subunits, five of which have direct orthologous relationship to a canonical CSN subunit: *Sc*Csn5 to Csn5, *Sc*Csn11 to Csn1, *Sc*Csn10 to Csn2, *Sc*Rpn5 to Csn4, and *Sc*Csn9 to Csn7 (Figure 2C, [29], [30], [31]). We focused our study on the sixth subunit, *Sc*Csi1, which does not bear any of the CSN-signature domains. By analyzing the amino acid sequences, we found that the carboxyl-terminal domain of *Sc*Csi1 shares homology with the C-terminal region of canonical Csn6 from multicellular organisms (Figure 1, 2A). This previously undefined domain is hereafter termed as *Csn6 C-terminal domain*, or S6CD. The S6CD is the most conserved region among orthologs of *Sc*Csi1 within the family of *Saccharomyces* (Figure S1).

Our observation that Csi1 orthologs exist only in organisms that lack canonical Csn6 raises the question whether Csi1 represents a functional substitute of Csn6 in *Saccharomyces*. To address this question, we have examined available open-genomes in the search for genomic orthologs of *CSN6*. Our results show that most organisms bear canonical version of Csn6 with both MPN and S6CD domains (Table S1). In several multicellular organisms, *CSN6* is either partially preserved, or absent (*Gallus gallus; Anolis carolinensiss*), possibly due to breaches in genomic sequence (Table S1). In addition, *CSN6* is not found in a completed genomic database of several parasitic protists including *Giardia intestinalis*, possibly due to a comprehensive loss of genes involve in protein quality control, including CSN encoding genes [32].

Through analyzing a collection of 75 completed genomes within the fungi kingdom, we identified canonical Csn6 with recognizable MPN^−^ and S6CD domains in most phyla, including basal fungal lineages such as chytrids, zygomycetes, basiomycetes and filamentous ascomyctes (Sure 1). Nevertheless, most species within the order of *Saccharomycetales* are systematically deviated to the extent that they no longer bear a recognizable homolog to canonical Csn6. Instead, a group of proteins similar to *Sc*Csi1 that harbor only S6CD, but not an MPN^−^ domain, emerges within several *Saccharomyces* species (Figure S1). An interesting example is found in the genomes of *Schizosaccharomyces* species, organisms of which (S.*pombe, S.octosporus, S.japonicus*) possess neither a recognizable homolog of Csn6 nor of Csi1. A putative Csn6 candidate, Csa1 (gi4467279, Figure 1), was reported by Liu et al 2003 [28], which exhibits borderline sequence similarity to PSMD7/Rpn8, the proteasome lid subunit paralogous to Csn6. Recently, the sequence database entry of *Sp*Csa1 was replaced by an amino-terminally extended version called *Sp*Mug166, which is unique to the *Schizosaccharomyces* group.

Unlike budding and fission yeast, a full version of the Csn6 gene encoding both MPN^−^ and S6CD domains exist in species within the *Candida* group, in which S6CD is more divergent compared to the MPN domain (Figure 1). An opposite situation was found in two species of the green unicellular algae *Ostreococcus*, so far the only clear example beside *Saccharomycetales*, that the conservation is confined to S6CD (Table S1). Interestingly in a few groups such as *Pichia,* homologs for Csn6 have not been detected, except for *Pichia stipitis*, which is closer to *Candida* than the other *Pichia* species (Figure 1) [33]. Not recognizing S6CD in other *Pichia* species may not necessarily rule out the existence of *CSN6/CSI1*-like genes, but identification of such a distant similarity would have to rely on experimental evidence. Overall, the data above suggests that orthologs of Csn6 are recognizable in most organisms; however, in many cases the MPN^−^ domain of these subunits is absent.

**Figure 3:**
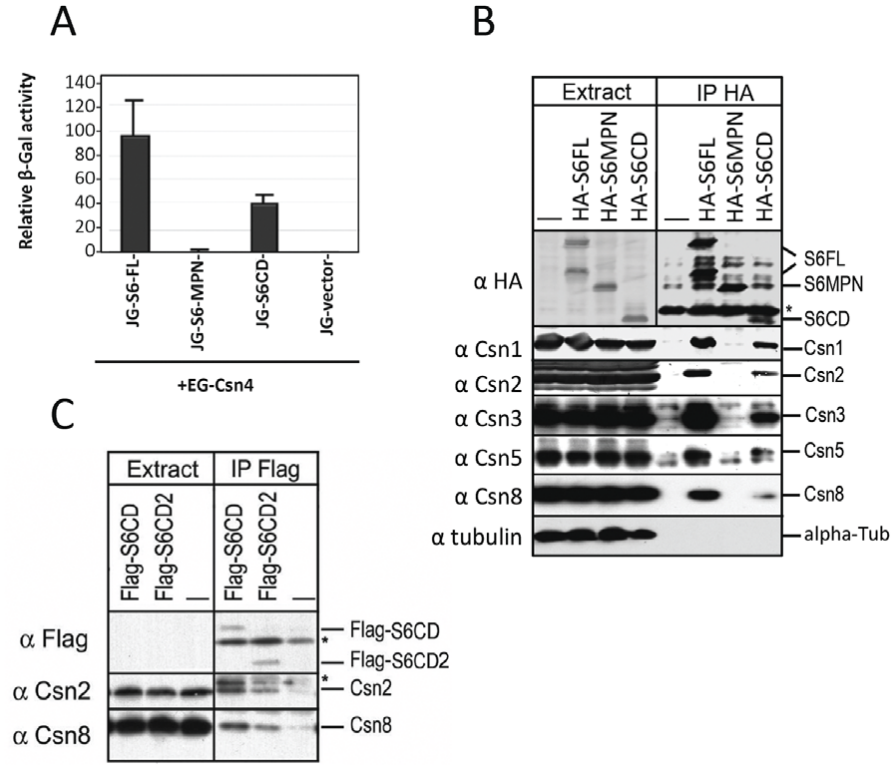
S6CD is necessary for the integrity of mammalian CSN complex. **A:** Yeast-2-hybrid assay showing interaction of mouse Csn4 (EG-Csn4) with Csn6 (JG-S6-FL) and the Csn6 C-terminal region (JG-S6CD). (See supplementary figure 2 for further information). **B:** S6CD region is necessary for integration of the protein into the CSN complex. HA-tagged full length mouse Csn6 (HA-S6FL) or the truncation mutants (HA-S6MPN and HA-S6CD) were expressed in HeLa cells. Cell lysates (left 4 lanes) and the HA (Convance Inc.) immunoprecipitated proteins (right 4 lanes) were analyzed by immunoblotting using specified antibodies as indicated on the right. **C:** Flag-tagged two C-terminal fragments of mouse Csn6 (S6CD, S6CD2) as described in Fig. 2B, were expressed in HEK293 cells. Proteins co-precipitated with the Flag-tag were blotted using indicated antibodies. Asterisk indicates IgG background. (**B, C**).

### S6CD is Sufficient, while MPN^−^ Domain is Dispensable, for CSN Complex Integrity

Considering that yeast CSN complex can carry out deneddylation of cullins without an MPN^−^ subunit [28], [29], [34], the role of MPN^−^ domain in the removal of Nedd8 from cullins becomes questionable. Using yeast-two-hybrid assay, a strong interaction between mouse Csn4 and Csn6 was detected (Figure S2), in agreement with previous published data (reviewed in [12], [35]). Similar experiment using individual truncation mutants of Csn6 demonstrate that Csn4 interacts with the C-terminal region of Csn6 that exclude most of the MPN^−^ domain, but not with the amino terminal part of Csn6 (Figures 2B, 3A).

We next expressed the HA tagged full length or different domains of Csn6 in mammalian cell lines (Figure 2B). Our results show that HA tagged Csn6 and S6CD, but not the MPN containing region (S6MPN) could co-precipitate endogenous Csn1–3, Csn5 and Csn8 (Figure 3B), indicating that the S6CD fragment can integrate into the complex with other CSN subunits. Similar results were obtained using Flag-tagged S6CD proteins, in which a small fragment of S6CD of 103 amino acid residues (Flag-S6CD2) was able to co-immunoprecipitate endogenous Csn2 and Csn8, albeit weaker than its longer version (Flag-S6CD) (Figure 2B, 3C). Therefore S6CD can assemble with other CSN subunits in vivo and is likely to play a role in structural integrity of the complex. In addition, Csn6 full length and S6CD, but not S6MPN, can co-immunoprecipitate Cul1 and Cul2, indicating that the C-terminal region of Csn6 is also necessary and sufficient for recruitment of cullins to the CSN complex, (Figure S3).

### Csn6 does not Form Homo-dimer in vivo

**Figure 4:**
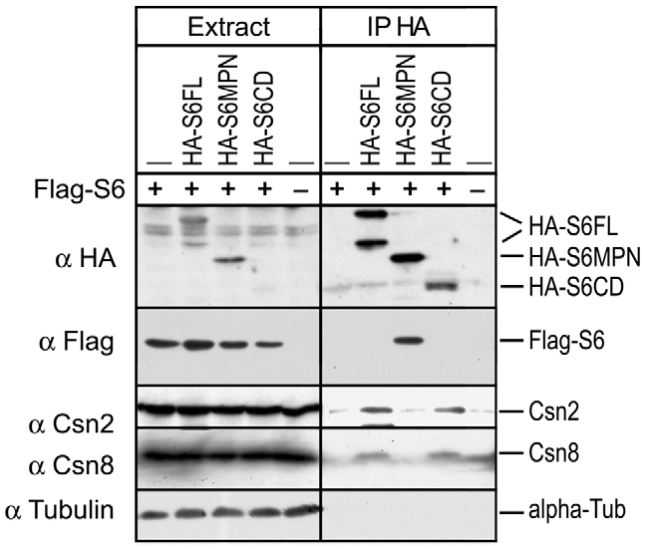
Only one copy of Csn6 is present in the CSN complex isolated via HA-S6FL or HA-S6CD. HA-tagged Csn6 constructs were co-expressed with a Flag-tagged full length Csn6, Flag-S6FL. Cell lysate (left 5 lanes) and HA (Bethyl S190-107) immunoprecipitated proteins were examined co-precipitation of Flag-S6FL or endogenous CSN subunits by immunoblotting using antibodies indicated on the right.

Structural studies of bacterially expressed MPN^−^ domain fragment of either Csn6 or its paraolg, Mov34/Rpn8, suggested that this domain exhibits in-vitro dimerization [36], [37]. To clarify the dimerization issue, we co-expressed Flag-Csn6 together with full length and truncation mutants of HA-Csn6. As shown in Figure 4, immunoprecipitation of HA-S6FL or HA-S6CD pulled down endogenous CSN subunits, but not the co-transfected Flag-S6. Interestingly, HA-S6MPN, which did not co-IP with endogenous CSN subunits, interacted with ectopically expressed Flag-Csn6 (Figure 4). These results show that the CSN complex bear only one copy of Csn6, either a full-length or a truncated form (S6CD); while the MPN^−^ domain fragment of Csn6, when expressed without the S6CD and incapable of integrating into the CSN complex, can still interact with over-stoichiometric amounts of Flag-Csn6 ([36], [38], Figure 3C). This result is consistent with reports on the dimerization of an MPN^−^ domain fragment of both Csn6 and Rpn8 [36], [38], but with an important clarification that the full length Csn6 does not dimerize in vivo. This result also confirms that the CSN complex pulled down by S6CD does not contain another copy of Csn6, and consequently, does not bear any MPN^−^ domain.

### The MPN^−^ Domain is not Required for CSN-mediated Deneddylation of Cullins

**Figure 5:**
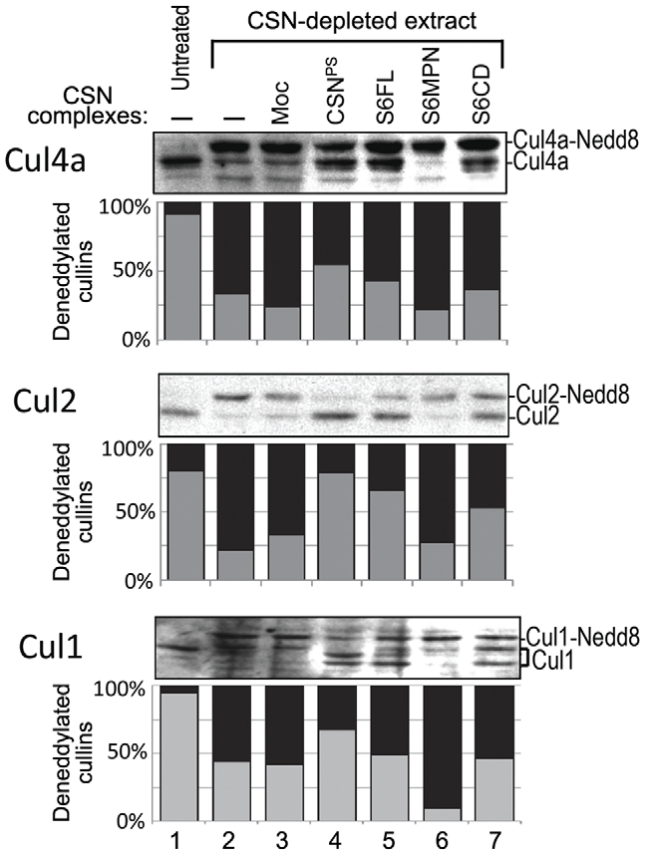
MPN^−^ domain of Csn6 is dispensable for CSN mediated cullin deneddylation. The deneddylation assays were performed using CSN-depleted cell extracts as a source of neddylated cullin substrates (lanes 2–7). The HA-purified complexes (S6FL, S6MPN, S6CD or mock) were tested for the deneddylation activity. Mock (Moc) was the HA purification from untransfected cells. Neddylation levels of Cul4a, Cul2, and Cul1 were examined by immunoblotting with respective antibodies. Normal extract (untreated) was taken before CSN-depletion. Biochemically purified porcine spleen CSN (CSN^PS^) was used as a positive control. Relative intensities of neddylated and deneddylated cullin protein bands were quantified using densitometry and imaging software IMAGEJ, taking into consideration of the local background. The values were presented as percentages of deneddylated cullins (gray bar, Y axis) or neddylated cullins (black bar) of the total amount of cullin proteins. The bracket in the Cul1 blot indicates the multiple deneddylation products that were taken into account for the quantification.

Having shown that the MPN^−^ domain is not required for CSN assembly, we next asked whether it is required for the deneddylase enzymatic activity of the complex. We obtained the CSN complex by one-step HA-affinity isolation of HA-Csn6 or the truncation derivatives from whole cells extracts under high salt, the condition that reduced co-purification of endogenous cullins (Figure S3). For substrate, we used CSN-depleted HeLa whole-cells extract, in which cullin proteins were predominantly in neddylated form (Figure S4) [ref 28, 29]. When HA affinity purified CSN complex was added to the extract, deneddylation occurred within 20 minutes as evidenced by the decline of neddylated Cul1, Cul2, and Cul4a and the increase of their deneddylated forms (Figure 5 lane 4). We found that the S6CD complex, which lacked Csn6 MPN^−^ domain, could effectively deneddylate Cul1, Cul2 and Cul4a, similar to S6FL (Figure 5 lanes 5 and 7). By contrast, the S6MPN pull-down (Figure 5 lane 6) exhibited only background level of deneddylation similar to the mock (Figure 5, lane 3), which were HA-beads pull-down from untransfected cells. These results showed that the MPN^−^ domain could be removed without compromising the deneddylation activity of multiple substrates by the CSN complex.

**Figure 6:**
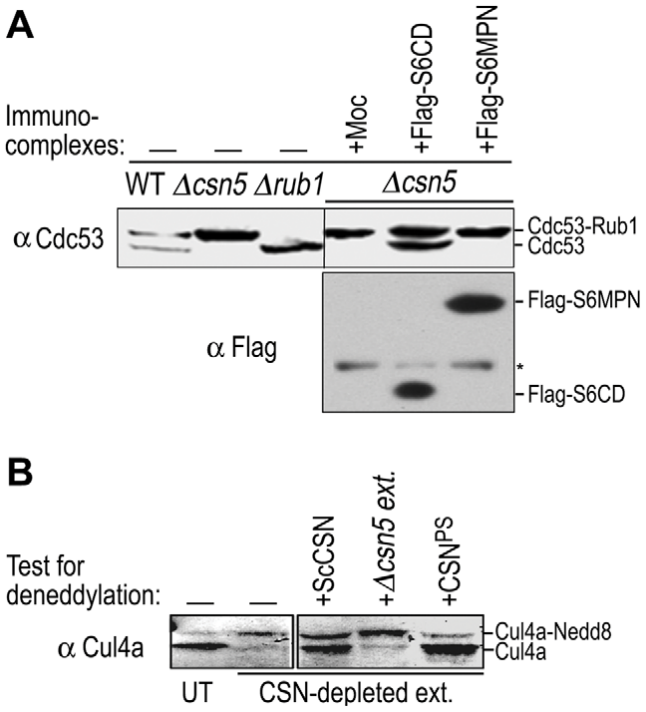
Deneddylation by yeast and mammalian CSN complexes that lacked the MPN^−^ domain on yeast or mammalian cullin substrates. A: The rubylated Cdc53 in Δ*csn5* extract was used as a substrate to test the deneddylation activity of Flag-purified mammalian complexes. Flag-S6CD, Flag-S6MPN, or empty vector were expressed in HeLa cells, isolated via the Flag beads, and eluted with the Flag peptide. The proteins were immunoblotted with indicated antibodies. Yeast cell extracts of wild type, Δ*rub1* and Δ*csn5* strains served as size markers for Cdc53 rubylated and un-rubylated forms. Asterisk indicates IgG background. B: Purified CSN complexes from yeast (ScCSN) or mammalian cells (CSN^PS^) were tested for the deneddylation activity on human Cul4a-Nedd8 conjugates in CSN-depleted HeLa cell extract. Δ*csn5* extract from yeast was used as a negative control. UT, untreated cell extract.

As a further confirmation, we performed a varied form of the deneddylation assay (Fig. 6A). Budding yeast Δ*csn5* extract, in which *Sc*Cdc53 (Cul1) is entirely rubylated (neddylated), was used as substrate, and Flag-peptide-eluted immunocomplexes that were associated with mammalian Flag-S6CD or Flag-S6MPN were tested for derubylation. Only Flag-S6CD, but not Flag-S6MPN, exhibited derubylation activity on *Sc*Cdc53, despite that Flag-S6MPN was present in higher level than Flag-S6CD (Fig. 6A). This result reinforced the finding that the MPN^−^ domain of Csn6 is not necessary for the deneddylase activity of the CSN complex.

### The Deviated *Sc*CSN Shares Functional Properties with the Canonical CSN Complex

Budding yeast represents the most deviated CSN complex both in subunit composition and in sequence homologies. Yet, like its counterpart in higher organisms, *Sc*CSN is responsible for deneddylation (or derubylation) of Cdc53, the yeast Cul1 equivalent [19], [30]. So far none of the canonical CSN subunits have been shown to complement *Sc*CSN subunits. Csn5 is the most conserved subunit, but expression of human Csn5/Jab1 in budding yeast cannot complement the Cdc53-derubylation defects of the Δ*csn5* mutant (data not shown). Similarly, we found that the derubylation defect of Δ*csi1* was not rescued when mouse Csn6 fragments were expressed in Δ*csi1* yeast cells (Fig. S5).

Since the MPN^−^ domain does not exist in *Sc*CSN, it clearly is not necessary for the enzymatic activity in *Sc*CSN mediated derubylation of Cdc53. However, this simple form of *Sc*CSN complex has not been tested on cullins of higher organism, although the 8-subunit containing mammalian CSN complex has been shown to deneddylate the yeast or plant cullins (Fig. 6A) [2], [30], [39]. Here we used a calmodulin affinity-purified *Sc*CSN complex to test on mammalian cullin substrates,. Remarkably, our results showed that the yeast CSN complex was able to deneddylate Cul4a in CSN-depeleted HeLa cell extract (Figure 6B). Together, our results show not only that the enzyme-substrate interplay of the CSN deneddylase is highly conserved between yeast and mammals, but also that deneddylation of mammalian cullins can be carried out by the budding yeast CSN, a simpler complex that lacks the MPN^−^ domain of Csn6. This result showed again that Csn6 MPN^−^ domain is not required for the deneddylase activity of the CSN complex.

## Discussion

### Toward Identification of the Core CSN Deneddylase

The CSN deneddylase centers at the JAMM motif of the Csn5 subunit, but is active only when assembled into a complex [17], [19]. While the enzymatic activity is evolutionarily conserved among all CSN orthologous complexes, the subunit numbers of the complex vary (Table 1, Figure 2C) [12], [29], [40]. This suggests that the canonical composition of the CSN complex, comprised of six PCI subunits and a pair of MPN^+^/MPN^−^ subunits, is not at all a prerequisite for its deneddylase function. By a comparative study of mammalian CSN with that of the budding yeast complex, which has, to date, the most simplified subunit composition, we have gained new insight about the minimal CSN “core” complex that is sufficient for this enzymatic activity.

The *S. cerevisiae* CSN is comprise of four PCI subunits (Csn1, Csn9, Csn10, Rpn5), an MPN^+^/JAMM subunit (Csn5), and Csi1 (Table 1, Figure 2C). Deletion of each of them abolishes the deneddylase activity, resulting in accumulation of rubylated Cdc53 in those mutants [19], [30]. Besides the conserved Csn5 subunit, the four budding yeast PCI subunits are believed to be equivalent to Csn1, Csn2, Csn4, and Csn7 of canonical CSN complex (Figure 2C). *Sc*Csi1 harbors an S6CD domain homologous to canonical Csn6 C-terminus (Figure 1, 2C), and we suggest that Csi1 is functionally equivalent to Csn6 (see below). It is still an open question whether Csn3 and Csn8 are part of the “core” CSN deneddylase, because the putative *S. cerevisiae* homologs of Csn3 and Csn8 (YPR045C/YJR084C; Sac3/Thp1) appear to have been separated from the CSN both structurally and functionally [12], [41]. Csn8 is also missing in *N. crassa* CSN, while Csn3 exists [42]. Although Csn3 co-purify with the CSN complex in *N. crassa*, its deletion does not result in a defect in deneddylation [42]. In plants and animals, deficiencies of Csn3 or Csn8 do affect deneddylation [43], [44], but that could be due to the roles of Csn3 and Csn8 in maintaining the structural stability of the CSN complex. Still, more research is needed to better understand how Csn3 and Csn8 may contribute to the deneddylase enzymatic activity.

### The MPN- Domain is Dispensable for CSN Assembly or Nedd8 Hydrolysis

Csn6 has been suggested to play an important role in maintaining structural stability of the CSN [22], and its deficiency disrupts CSN complex in plants [43]. Likewise, Rpn8, which is the corresponding paralog of Csn6 in the lid, plays a critical role in maintaining the lid configuration by spanning the horseshoe-like structure made up of the PCI subunits [13].

Csn6 directly interacts with Csn4 and Csn7 (Figure S2) [1], [21], [22], while in parallel; *Sc*Csi1 interacts with *Sc*Rpn5 and *Sc*Csn9 [45], the yeast equivalents of Csn4 and Csn7 (Figure 2C). At least with mammalian Csn6, the C-terminal region containing the S6CD is necessary and sufficient for the assembly of the CSN complex (Figure 3, 4). Moreover, both the mammalian CSN complex assembled with S6CD and the yeast CSN complex, lacking the MPN^−^ domain, were active in deneddylation of human cullins (Figures 5, 6). Thus, our data demonstrate that the MPN^−^ domain is not required for either complex assembly, nor is it required for the deneddylase activity of the complex, and the loss of MPN^−^ domain in several *Ascomycota* without losing the deneddylase activity is in full agreement with our findings.

### What is the Function of the MPN- Domain in Csn6?

To date, a common biological paradigm of mechanistic role for MPN^−^ proteins, if there is, has not been revealed. Like the CSN and lid, BRCA1-A and BRISC deubiquitinating complexes contain a pair of an MPN^−^ and MPN^+^/JAMM proteins (Table 1). Interaction between the two MPN proteins has been reported [46]. It has been speculated that the MPN^−^ domain may support the MPN^+^ catalytic subunit by orienting the ubiquitin chain to allow efficient cleavage [46], although this theory has not been tested. MPN^−^ proteins have been found also in protein complexes without MPN^+^/JAMM proteins such as U5 snRNP spliceosome (Prp8) and the eIF3 complex (eIF3f and eIF3h) [47]–[48]. In Prp8, the MPN^−^ domain locates at the carboxyl terminal region, and it can bind ubiquitin with an affinity comparable to other known ubiquitin binding domains [48]. It was suggested that MPN^−^ domain of Prp8 represents a “pseudoenzyme” that has lost its enzymatic activity, but has preserved enzyme’s affinity to its substrate [48]. Two MPN- subunits are found within the eIF3 complex, among them eIF3f exhibits deubiquitinating activity in-vitro, and a catalytically inactive mutant leads to accumulation of monoubiquitinated Notch in-vivo [49].

The recent cryo-electron microscopy of the Rpn8 structure in the lid provides an informative picture on this MPN^−^ protein. It is noted that Rpn8, as well as the MPN^+^/JAMM subunit Rpn11, undergo significant conformational changes upon integration of the lid into the proteasome holoenzyme, which simultaneously activates the JAMM DUB activity of the proteasome [13]. It seems plausible that some of the interactions involving these MPN subunits in the free lid might act to inhibit the enzymatic activity prior to the holocomplex assembly. Obviously, CSN’s activity is not regulated by its recruitment into a proteasome-like complex, but it is regulated nonetheless. For example, CSN inhibits some CRL4 ubiquitin ligases, but in a manner dependent on UV damage [50], [51]. However, the precise mechanism of such regulation is yet unknown.

Conservation of the Csn6 MPN^−^ domain among higher organisms may suggest that it has a function related to CSN regulation in context with the developmental or physiological processes of complex organisms. Csn6 MPN^−^ domain has been shown to mediate interaction with the14-3-3σ protein, but whether and how the CSN is involved is unclear [52]. Clearly, extensive experimentations are needed to understand the specific function of the MPN^−^ domain of CSN6 in the COP9 signalosome.

## Materials and Methods

### Strains and Cell Growth Conditions

Yeast cells were grown under standard growth conditions at 28°C. Wild-type, *CSN5* deletion or a genomic Csn5-TAP tag strains were purchased from EUROSCARF (Frankfurt, Germany). HeLa and HEK293 cells were cultured and transfected as previously documented [53].

### Plasmids

All plasmids are listed in Table S2. Mouse cDNA was used as a template for the subcloning of Csn6 full length (FL) or truncation mutants (MPN, S6CD, S6CD2) into mammalian expression vectors and the yeast Yeplac181 vector.

### Antibodies

The following antibodies were used in this study: anti-Csn1, anti-Csn2, anti-Csn8 [54], anti-Csn6 (Aviva systems biology); anti-Cul1, anti-Cul2, anti-Cdc53 (Santa Cruz Biotechnology, Santa Cruz, CA); anti-Cul4 [53]; anti-Flag (antibody and agarose beads),anti-alpha tubulin, anti-HA antibody (Sigma), HA beads (Bethyl and Convance).

### Yeast Two-hybrid and Immunoblots

The yeast two-hybrid liquid assay measuring relative ß-galactosidase activity of the reporter plasmid (pSH18-34) was performed as previously described [14]. For immuno blotting, yeast cells were cultured overnight at 30°C. Cells were harvested, washed twice with double-distilled water, and protein extract was prepared according to Yu et al. 2011 [29].

### Purification of CSN Complexes

*Sc*CSN complex for figure 6B was purified by one step of purification through a CBP (Calmodulin-binding peptide) tag on calmodulin agarose beads (GE) in yeast cell expressing Csn5-TAP, according to Yu et al. 2011 [29]. HA and Flag purifications of transfected cells or untransfected cells (mock), were performed, as previously described [53], [55]. After purification, part of HA-beads-bound complexes or mock were used for immunoblotting, and part were used for deneddylation assay. For Flag based purification, complexes were eluted from the beads with 1mM of Flag peptide as previously described [29]. Conventional purification of porcine spleen CSN complex was performed according to Menon et al. 2005 [44]. Note that Porcine S6CD is identical to the human and mouse ortholog domain (Figure 2B).

### Deneddylation Assays

CSN-depleted HeLa cell extract [44], [56] were used as source of neddylated cullin substrates. The CSN complexes to be tested for deneddylation were obtained by HA or Flag immunoprecipitation under high salt condition (300mM NaCl) from cells expressing HA- or Flag-tagged Csn6 or the mutants. Protein extraction and affinity purification has been described previously. One third of washed Csn6 immuno-complexes bound to HA beads were analyzed by western blotting to check for successful IP, and two thirds of the beads were used for the deneddylation assay. Each sample was mixed with an equal amount of CSN-depleted extract, and the reaction was incubated for 20 min at 30°C. The reaction was stopped by adding Laemmli sample buffer. Status of cullin neddylation was examined by anti-cullin immunoblots. The ratio between neddylated and un-neddylated cullins was analyzed using densitometry and imaging software IMAGEJ (http://rsbweb.nih.gov/ij/). In the experiment shown in figure 6A, Flag-S6MPN and Flag-S6CD bound immunocomplexes were eluted by 1mM of Flag peptide in elution volume of 150 µl. 40 µl of each of the eluted complexes were added to each experimental tube in addition to equal amount of Δ*csn5* cells extract. Derubylation of Cdc53-Rub1 was performed as previously documented [29]. Experiments were repeated 3 times, and a representative result is shown.

## Supporting Information

Figure S1 **Orthologs of Csi1 within** ***Saccharomyces*** **.** A. Direct orthologs for Csi1 are found within 9 Ascomycete fungal genomes. A ClustalW alignment of the *S. cerevisiae* Csi1 with identified orthologs in other fungal species. B. Sequence-based gene tree. Figures display information according to the Fungal Orthogroups Repository website: http://www.broadinstitute.org/regev/orthogroups/.
(DOCX)

Figure S2 **Yeast two-hybrid pair wise interactions between Csn6 and other CSN subunits.** Full-length mouse Csn6 was expressed as a LexA DNA binding domain (LexACSN1) fusion protein and other subunits of the mouse COP9 signalosome were expressed as transcription activation domain (AD-CSNs) fusion proteins (Golemis et al., 1994). Pair-wise interactions were indicated by relative beta-galactosidase activities of the reporter plasmid (pSH18-34). The values were relative to the positive control, pSH17-4 (at 100) (LexA-AD fusion). Six independently transformed samples were used to calculate the averages and the standard deviations (error bars). In all samples, protein expression for the respective construct was confirmed by immunoblotting using anti-LexA (Clontech) and anti-HA antibodies (Santa Cruz).
(DOCX)

Figure S3 **HA-tagged Csn6 and truncation mutants were used in co-immunoprecipitation in HeLa cell extract to map interactions with cullins.** Full length Csn6 (3HA-S6FL) and the S6CD fragment, but not the MPN domain, could co-immunoprecipitate Cul1 and Cul2. Note that addition of 330 mM NaCl to the binding buffer interfered with CSN-cullins interactions.
(PDF)

Figure S4 **Deneddylation assay. CSN-depleted HeLa cell extracts were used as a source for neddylated cullin substrates, as compared to untreated (UT) extract.** These neddylated cullins could be effectively deneddylated by the CSN complex purified from porcine spleen (CSN^PS^). The reaction mixtures were western blotted using anti-cullin antibodies. The amounts of CSN were detected by immunoblotting with anti-Csn1 and ant-Csn2 antibodies.
(PDF)

Figure S5 **Fragments of mouse Csn6 cannot complement derubylation defects of Δ** ***csi1*** **mutant of the budding yeast.** Truncated fragments of mouse Csn6 were ectopically expressed in WT or Δ*csi1* yeast strains. Complementation of derubylation by Csi1 was confirmed as well (brackets stand for over-expression). Total cell extracts were used for western blot analysis of Cdc53. Expression of Csn6 proteins was determined by immunoblotting with anti-Flag, and with anti-Csn6, which recognizes antigenic peptide of AA150–200 that is present only in CBP-Flag-S6CD.
(PDF)

Table S1 **Bioinformatic identification of two distinct Csn6 domains, in non-fungal organisms.** Canonical Csn6, including both MPN- and S6CD domains, is found in most organisms. The absence of Csn6 in a few organisms could be due to fractions in genome sequences. Interestingly, a few protozoans appear to be devoid of all CSN genes including *Csn6*. Two unicellular species of the green algae *Ostreococcus* contain Csn6 with a conserved S6CD and a deviated MPN- domain.
(DOCX)

Table S2 **List of plasmids used in this study.**
(DOCX)
