# Supplementary material for: The Minimal Deneddylase Core of the COP9 Signalosome Excludes the Csn6 MPN− Domain
Source: PLoS One. 2012 Aug 30;7(8):e43980. doi: 10.1371/journal.pone.0043980 (PMC3431379; doi:10.1371/journal.pone.0043980)
Supplement: Figure S1 — Orthologs of Csi1 within Saccharomyces . A. Direct orthologs for Csi1 are found within 9 Ascomycete fungal genomes. A ClustalW alignment of the S. cerevisiae Csi1 with identified orthologs in other fungal species. B. Sequence-based gene tree. Figures display information according to the Fungal Orthogroups Repository website: http://www.broadinstitute.org/regev/orthogroups/. (DOCX) [file pone.0043980.s001.docx]

****Figure S1:

B

A
