# Supplementary material for: The Minimal Deneddylase Core of the COP9 Signalosome Excludes the Csn6 MPN− Domain
Source: PLoS One. 2012 Aug 30;7(8):e43980. doi: 10.1371/journal.pone.0043980 (PMC3431379; doi:10.1371/journal.pone.0043980)
Supplement: Figure S2 — Yeast two-hybrid pair wise interactions between Csn6 and other CSN subunits. Full-length mouse Csn6 was expressed as a LexA DNA binding domain (LexACSN1) fusion protein and other subunits of the mouse COP9 signalosome were expressed as transcription activation domain (AD-CSNs) fusion proteins (Golemis et al., 1994). Pair-wise interactions were indicated by relative beta-galactosidase activities of the reporter plasmid (pSH18-34). The values were relative to the positive control, pSH17-4 (at 100) (LexA-AD fusion). Six independently transformed samples were used to calculate the averages and the standard deviations (error bars). In all samples, protein expression for the respective construct was confirmed by immunoblotting using anti-LexA (Clontech) and anti-HA antibodies (Santa Cruz). (DOCX) [file pone.0043980.s002.docx]

Figure S2:
