# Supplementary figures and images for: The Minimal Deneddylase Core of the COP9 Signalosome Excludes the Csn6 MPN− Domain
Source: PLoS One. 2012 Aug 30;7(8):e43980. doi: 10.1371/journal.pone.0043980 (PMC3431379; doi:10.1371/journal.pone.0043980)

Figure S3:

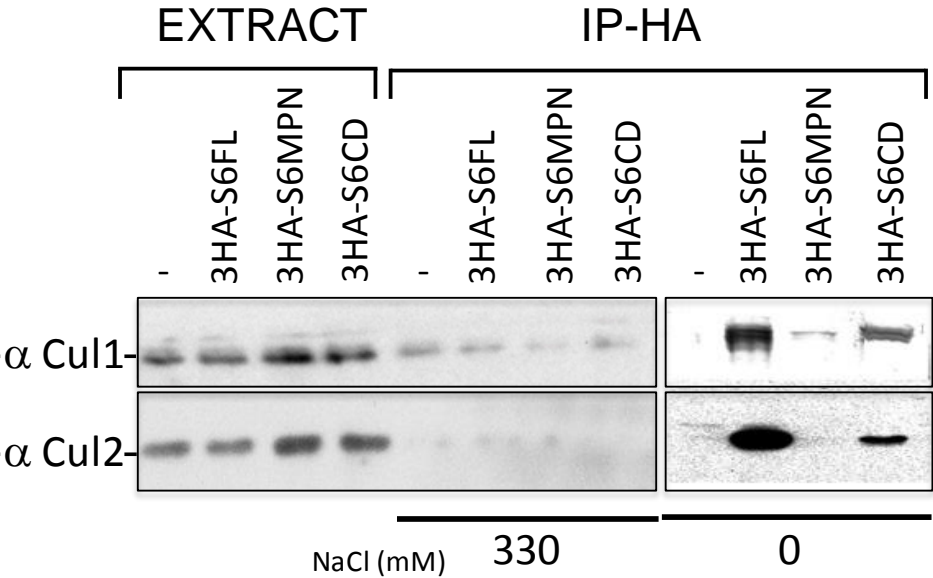

Supplement: Figure S3 — HA-tagged Csn6 and truncation mutants were used in co-immunoprecipitation in HeLa cell extract to map interactions with cullins. Full length Csn6 (3HA-S6FL) and the S6CD fragment, but not the MPN domain, could co-immunoprecipitate Cul1 and Cul2. Note that addition of 330 mM NaCl to the binding buffer interfered with CSN-cullins interactions. (PDF) [file pone.0043980.s003.pdf]

Figure S4

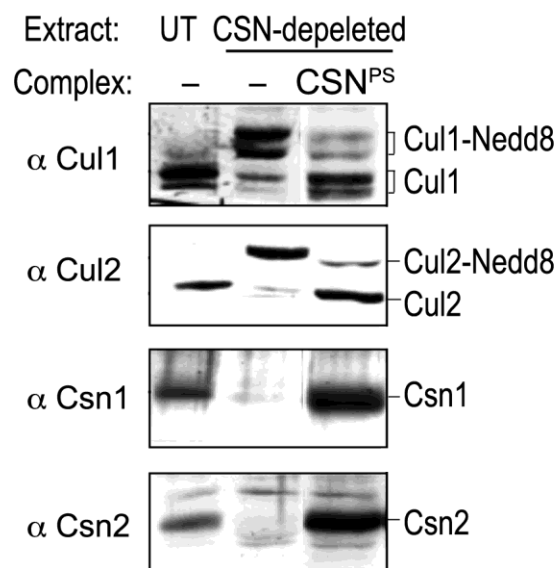

Supplement: Figure S4 — Deneddylation assay. CSN-depleted HeLa cell extracts were used as a source for neddylated cullin substrates, as compared to untreated (UT) extract. These neddylated cullins could be effectively deneddylated by the CSN complex purified from porcine spleen (CSNPS). The reaction mixtures were western blotted using anti-cullin antibodies. The amounts of CSN were detected by immunoblotting with anti-Csn1 and ant-Csn2 antibodies. (PDF) [file pone.0043980.s004.pdf]

Figure S5:

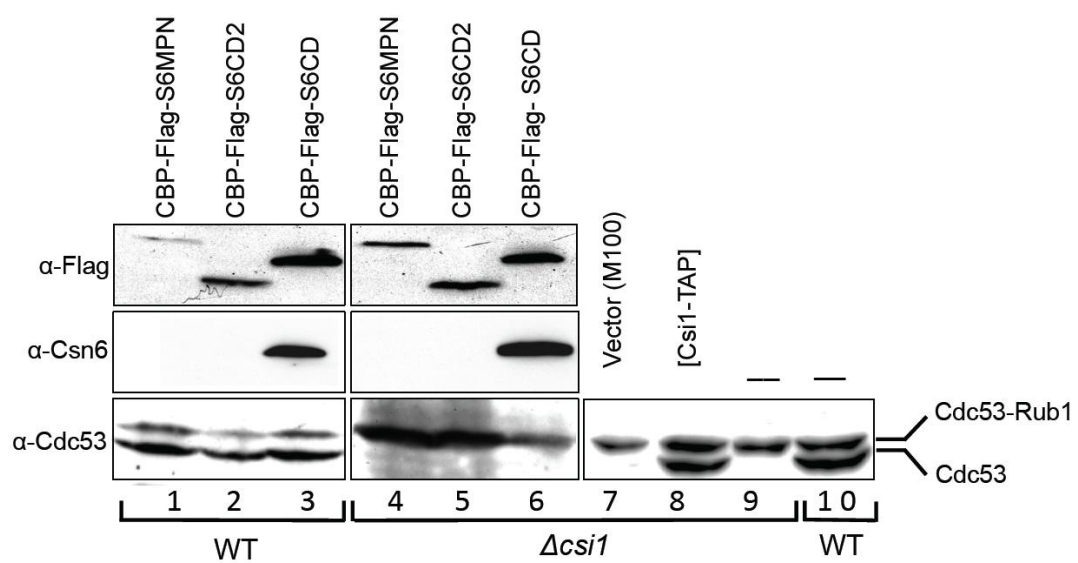

Supplement: Figure S5 — Fragments of mouse Csn6 cannot complement derubylation defects of Δ csi1 mutant of the budding yeast. Truncated fragments of mouse Csn6 were ectopically expressed in WT or Δcsi1 yeast strains. Complementation of derubylation by Csi1 was confirmed as well (brackets stand for over-expression). Total cell extracts were used for western blot analysis of Cdc53. Expression of Csn6 proteins was determined by immunoblotting with anti-Flag, and with anti-Csn6, which recognizes antigenic peptide of AA150–200 that is present only in CBP-Flag-S6CD. (PDF) [file pone.0043980.s005.pdf]
