# Supplementary material for: The Minimal Deneddylase Core of the COP9 Signalosome Excludes the Csn6 MPN− Domain
Source: PLoS One. 2012 Aug 30;7(8):e43980. doi: 10.1371/journal.pone.0043980 (PMC3431379; doi:10.1371/journal.pone.0043980)
Supplement: Table S2 — List of plasmids used in this study. (DOCX) [file pone.0043980.s007.docx]

Table S2:

| **Plasmid name** | **Insert** | **Parental vector** | **Expression system** | **Source** |
| --- | --- | --- | --- | --- |
| JG-S6-FL | *CSN6*1-324 | pJG4-5 , for Y2H | *S. cerevisiae* | This study |
| JG-S6-MPN | *CSN6*1-139 | pJG4-5 , for Y2H | *S. cerevisiae* | This study |
| JG-S6-CTD | *CSN6*127-324 | pJG4-5 , for Y2H | *S. cerevisiae* | This study |
| JG-vector | JG-vector | pJG4-5 , for Y2H | *S. cerevisiae* | This study |
| EG-Csn4 | *CSN4* | pEG202, for Y2H | *S. cerevisiae* | This study |
| 3HA-S6-FL | 3HA-S6-FL | pcDNA3.3HA | HeLa, HEK293 cells | This study |
| 3HA-S6-MPN | 3HA-S6-MPN | pcDNA3.3HA | HeLa, HEK293 cells | This study |
| 3HA-S6-CTD | 3HA-S6-CTD | pcDNA3.3HA | HeLa, HEK293 cells | This study |
| EP50 | Flag-S6-MPN | pCMV-FLAG | HeLa, HEK293 cells | This study |
| EP81 | Flag-S6-LCTD | pCMV-FLAG | HeLa, HEK293 cells | This study |
| EP99 | Flag-S6-SCTD | pCMV-FLAG | HeLa, HEK293 cells | This study |
| EP2 | Csn6-MPN | Yeplac181, ADHp, ADHt | *S. cerevisiae* | This study |
| EP3 | S6CD1 | Yeplac181, ADHp, ADHt | *S. cerevisiae* | This study |
| EP4 | S6CD2 | Yeplac181, ADHp, ADHt | *S. cerevisiae* | This study |
| M100 |  | pYES2, GAL1p | *S. cerevisiae* | Invitrogene |
| EP88 | Csi1-TAP | BG1805 | *S. cerevisiae* | Open Biosystems |

**Table S2:** List of plasmids used in this study
